# Editorial: Understanding the deviant behavior in workplace: formation mechanism, impacts, and consequences

**DOI:** 10.3389/fpsyg.2023.1252105

**Published:** 2023-08-07

**Authors:** Jintao Lu, Krisztina Szegedi, Jiaojiao Qu, Guanglei Zhang, Jianfeng Jia, Xiaoguang Liu

**Affiliations:** ^1^School of Economics and Management, Taiyuan University of Science and Technology, Taiyuan, China; ^2^Research Center for Corporate Social Responsibility, Taiyuan University of Science and Technology, Taiyuan, China; ^3^Budapest Business School, Budapest, Hungary; ^4^Business School, Huaqiao University, Quanzhou, China; ^5^School of Management, Wuhan University of Technology, Wuhan, China; ^6^School of Business Administration, Northeastern University, Shenyang, China; ^7^College of Economics and Management, Taiyuan University of Technology, Taiyuan, China

**Keywords:** deviant behavior, workplace, organizational management, decision psychology, work performance

In recent years, due to the impact of the COVID-19 pandemic and economic downtrend, organizations are facing more serious challenges along with the increasing uncertainty of the organizational environment, and the globalization, migration movement, and cultural integration, and individuals are also encountering higher pressures from work performance and life difficulties, deviant behavior in organizational management has become a hot topic in academic community. In order to explore the antecedents and consequences of deviant behaviors from the perspectives of cognition, morality, and decision psychology, etc., we set this Research Topic to cover a wide range of themes related to “Deviant Behavior in the Workplace”, hoping related conclusions can help organizations better manage such behaviors and meet future management challenges.

In this Research Topic, we totally received 22 submissions from distinguished scholars all over the world, after strict peer review we finally accepted and published 8 articles, the acceptance rate is 36.4%. In the first article by Zhang et al., this study proposed a moderated mediation model of authoritarian leadership on subordinates' cyberloafing via Paired samples of 360 employees working in 103 teams from Chinese companies were collected at 2 points in time. The findings expanded the literature on authoritarian leadership and cyberloafing and had significant practical implications for managing employees in this digital era. In the second article by Qin and Zhang, it was dedicated to exploring the influence of perceived overall injustice on employee anger and deviant behavior using three Chinese manufacturing corporations with a total effective sample size of 264. The obtained conclusions provided empirical evidence to overall injustice literature, supplement workplace emotion research, and enrich social cognitive studies. In the third article by Zhao and Qu, it explored the underlying relationship between employees' unethical pro-organizational behavior (UPB) and their own pro-environmental behavior based on the conservation of resources theory, which provided empirical evidence to analyze how UPB affects employees' cognitions, attitudes, and behaviors. In the fourth article by Chen et al., it focused on the influences of (in)congruences in psychological entitlement and felt obligation on employees' (un)ethical behavior using 202 matched questionnaires from full-time Chinese workers. The findings provided a novel insight into the interactive influences of (in)congruence in psychological entitlement and felt obligation on employees' ethical behavioral choices. In the fifth paper by Hou et al., drawing on personal identification theory, this study explored the questions of whether, why, and when unethical pro-family behavior (UPFB) could be inhibited by leader self-sacrificial behavior (LSSB). In the sixth article by Jia et al., via three-wave time-lagged surveys to 251 employees, it followed the stimuli-organism-response model and used psychological ownership theory to examine a moderated mediation model with psychological ownership as a mediator and Chinese traditionality as a moderator to interpret how and when high-involvement human resource management practices influence employees' bootlegging. In the seventh article by Ni et al., it emphasized the study of impression management tactic from the perspective of within-individual, but also integrates the perspective of between-individuals by employing the survey method of job logs and collects the data of 121 employees, which breaks through the between-individual perspective and illustrates the double-edged sword effect of self-promotion tactic and ingratiation tactic on employee counterproductive work behavior and its mechanism from the internal perspective. Finally in the eighth article by Gao et al., by integrating social identity theory with social information processing theory, this study explained when supervisors perceived subordinate unethical pro-organizational behavior (UPB) in a negative way, and further engaged in negative leading behaviors as punishments for UPB. The obtained findings extends understanding of when and why supervisors punish rather than indulge subordinates who act in ethically questionable ways and provide important insights into supervisors' leading behavior from a bottom-up perspective.

We thank all the involved reviewers and editors who have been contributing to the release of this Research Topic by their devoted engagement. We herewith also wish the readers of the journal can enjoy reading these 8 articles as we enjoyed and editing this issue.

## Author contributions

All authors listed have made a substantial, direct, and intellectual contribution to the work and approved it for publication.

